# Loss of *Dmrt5* Affects the Formation of the Subplate and Early Corticogenesis

**DOI:** 10.1093/cercor/bhz310

**Published:** 2019-12-16

**Authors:** Leslie Ratié, Elodie Desmaris, Fernando García-Moreno, Anna Hoerder-Suabedissen, Alexandra Kelman, Thomas Theil, Eric J Bellefroid, Zoltán Molnár

**Affiliations:** 1 ULB Neuroscience Institute, Université Libre de Bruxelles, B-6041 Gosselies, Belgium; 2 Department of Physiology, Anatomy and Genetics, University of Oxford, Oxford OX1 3PT, UK; 3 Achucarro Basque Center for Neuroscience, Parque Científico UPV/EHU Edif. Sede, E-48940 Leioa, Spain; 4 IKERBASQUE Foundation, 48013 Bilbao, Spain; 5 Centre for Discovery Brain Sciences, University of Edinburgh, Edinburgh EH8 9XD, UK

**Keywords:** corticogenesis, Dmrt, neuronal migration, subplate

## Abstract

*Dmrt5* (*Dmrta2*) and *Dmrt3* are key regulators of cortical patterning and progenitor proliferation and differentiation. In this study, we show an altered apical to intermediate progenitor transition, with a delay in SP neurogenesis and premature birth of Ctip2^+^ cortical neurons in *Dmrt5*^−/−^ mice. In addition to the cortical progenitors, DMRT5 protein appears present in postmitotic subplate (SP) and marginal zone neurons together with some migrating cortical neurons. We observed the altered split of preplate and the reduced SP and disturbed radial migration of cortical neurons into cortical plate in *Dmrt5*^−/−^ brains and demonstrated an increase in the proportion of multipolar cells in primary neuronal cultures from *Dmrt5*^−/−^ embryonic brains. Dmrt5 affects cortical development with specific time sensitivity that we described in two conditional mice with slightly different deletion time. We only observed a transient SP phenotype at E15.5, but not by E18.5 after early (*Dmrt5*^*lox/lox*^*;Emx1*^*Cre*^), but not late (*Dmrt5*^*lox/lox*^*;Nestin*^*Cre*^) deletion of Dmrt5. SP was less disturbed in *Dmrt5*^*lox/lox*^;*Emx1*^*Cre*^ and *Dmrt3*^−/−^ brains than in *Dmrt5*^−/−^ and affects dorsomedial cortex more than lateral and caudal cortex. Our study demonstrates a novel function of *Dmrt5* in the regulation of early SP formation and radial cortical neuron migration.

**Summary Statement:**

Our study demonstrates a novel function of *Dmrt5* in regulating marginal zone and subplate formation and migration of cortical neurons to cortical plate.

## Introduction

The mechanisms that control progenitor proliferation and differentiation are pivotal for correct cortical cell number and diversity. As such, corticogenesis is regulated by an array of transcription factors regulating progenitor self-renewal, differentiation, and death (Rakic 1988). A family of them are the Dmrts (doublesex and mab-3-related-transcription factor), which received their name from the two proteins doublesex (dsx) in *Drosophila melanogaster* and mal abnormal (mab-3) in *Caenorhabditis elegans* (Erdman and Burtis 1993; Johnsen and Andersen 2012). Members of this gene family encode proteins characterized by the presence of a cystein-rich DNA-binding motif known as the DM domain (Zhu et al. 2000). DMRT proteins have been classified into distinct subgroups, based on the presence of additional conserved protein domains. DMRT3, DMRT4, and DMRT5 (also designated DMRTA2) constitute one such subgroup characterized by the presence of a conserved DM domain (Bellefroid et al. 2013; Konno et al. 2012). *Dmrt3* and *Dmrt5* are both expressed in cortical apical progenitors of the developing cortex in a similar high caudomedial to low rostrolateral gradient, which is opposite to *Dmrt4* expression (Bellefroid et al. 2013).

*Dmrt5* and *Dmrt3* are an integral part of the genetic cascade that controls the development of the cerebral cortex. It has been suggested that *Dmrt5* and *Dmrt3* are required for proper cortical development and cooperatively control the expression of some proneural genes, cell cycle regulators, key transcriptional regulators of cortical patterning, and progenitor proliferation and differentiation (De Clercq et al. 2018; Desmaris et al. 2018; Konno et al. 2012; Saulnier et al. 2013). In human, a loss-of-function mutation in DMRT5 (DMRTA2) has been associated with microcephaly (Urquhart et al. 2016). However, the *Dmrt5* gene is also expressed in postmitotic neurons, but little is known about its specific functions at these later stages of cortical development, especially in subplate (SP).

SP neurons (SPns) are a heterogeneous population of cortical neurons with diverse developmental origin. They are among the earliest born neurons during embryonic development and play a fundamental role in the establishment of intra and extracortical circuits (Allendoerfer and Shatz 1994; Molnár et al. 1998; Hoerder-Suabedissen and Molnár 2015; Kostovic and Rakic 1990, 1980). SPns are present in a large number in the developing brain and are key for the functional maturation of the cerebral cortex, but after completion of the cortical circuit assembly, a large proportion of them disappear by preferential cell death and only few remain as interstitial white matter cells or layer 6b by adulthood (McConnell et al. 1989; Allendoerfer and Shatz 1994; Price et al. 1997).

Little is known about the neurogenesis and migration of SPn. In mouse, SPns are generated between E10.5 and E12.5 stages and initially contribute to the preplate (PP) (Price et al. 1997). Subsequently, the PP is split into marginal zone (MZ) and SP by the successive waves of migratory cortical neurons that start to occupy their position in the cortical layers in an inside-first outside-last pattern (Götz and Huttner 2005; Marin-Padilla 1971; Paridaen and Huttner 2014; Rakic 1978). SP provides a platform for the thalamocortical projections to accumulate and start to establish the earliest circuits while the cortical plate (CP) is constructed (Allendoerfer and Shatz 1994; Kanold and Luhmann 2010; Molnár et al. 1998). Migration through and the interactions with SP are now considered a vital part of cortical development and interactions between SP, and cortical migrating neurons are involved in the cell fate determination of CP neurons (Ohtaka-Maruyama et al. 2018; Ozair et al. 2018). Failure of normal cortical neuron migration can lead to aggregates in unusual areas (heterotopias), which are the characteristic of cerebral disorders such as lissencephaly and double cortex syndrome (Dobyns and Das 1993; Olson and Walsh 2002). Abnormal development of the earliest cortical circuits involving SPns has been described in a mouse model of autism spectrum disorder (Nagode et al. 2017). Alterations in the distribution and number of interstitial white matter neurons, which are considered as the remnant of SPns, have also been reported in schizophrenia and autism spectrum disorder (Akbarian et al. 1996, 1993; Connor et al. 2011; Kostović et al. 2011; Serati et al. 2019).

To understand the role of *Dmrt5*, we studied the proportion of various cortical progenitors, birth dated the waves of the earliest born neurons of the cortex, examined the split of PP into MZ and SP, and CP formation in developing *Dmrt5*^−/−^ mouse brains. Our study demonstrates that in addition to the *Dmrt5* transcription in apical progenitors, DMRT5 immunoreactivity is also detectable in some SPn, MZ neurons, and some migrating CP neurons. In *Dmrt5*^−/−^ mice, the differentiation of apical and intermediate progenitors is affected and this leads to the early disorganization of SP and CP. The timing and sequence of early-born neuron [SPn and deep layer neurons (dLns)] generation are shifted. Analysis of neuronal morphology in dissociated cortical cultures revealed an increase in the proportion of multipolar neurons, consistent with the altered radial neuronal migration observed in the *Dmrt5*^−/−^ mice. Altogether, our study demonstrates novel functions of *Dmrt5* in the regulation of SP formation and migration of cortical neurons to CP.

## Material and Methods

### Mouse Strains/Animals

All mouse experiments were conducted according to national and international guidelines and have been approved by the local ethics committee (LA1500474) and/or in accordance with the Animals (Scientific Procedures) Act, 1986 (ASPA), UK, under valid personal and project licenses.

*Dmrt5* ^−/−^, *Dmrt3*^−/−^, *Dmrt5^lox/lox^*;*Emx1Cre*, *Dmrt5^lox/lox^*;*NestinCre*, and *Dmrt5*^*tg*/tg^;*Emx1Cre* mutant mice were maintained on a C57BL6/J background. Heterozygous *Dmrt5*^+/−^ mice were obtained and incrossed in order to study the phenotype of embryos. PCR genotyping was performed as previously described (Desmaris et al. 2018). The morning of the vaginal plug was considered embryonic day (E) 0.5. Littermate embryos served as controls for all experiments.

*Lpar1-GFP* males (Tg(Lpar1-EGFP)GX193Gsat) were mated with wild-type (*WT*) NIHS females and maintained in an NIHS background (Hoerder-Suabedissen et al. 2013).

### *In situ* Hybridization and Immunofluorescence

For *in situ* hybridization (ISH), embryonic brains were dissected in phosphate buffered saline (PBS) and fixed overnight at 4 °C in 4% paraformaldehyde (PFA) in PBS. Brains were dehydrated and cryoprotected overnight in 30% sucrose and frozen in gelatine 7.5%-sucrose 15% in PBS. Brains were cryosectioned in the coronal plane on Leica CM1850© cryostat (25 μm). ISH experiments were performed as previously described (Desmaris et al. 2018).

The antisense probes were generated from the following previously described cDNA clones, *Nurr1* (Hasenpusch-Theil et al. 2012), *Tbr1* (Bedogni et al. 2010), *Pcp4*, and *Pls3* (Oeschger et al. 2012); *Ctgf* (Hoerder-Suabedissen et al. 2009); and *Reelin* (Yoshida 2006). ISH was performed as previously described (Desmaris et al. 2018). ISH images were acquired with an Olympus SZX16© stereomicroscope and a XC50 camera using CellSens Imaging© software. Images for publication were contrast adjusted and compiled using Adobe Photoshop CS3©.

IF and IHC experiments were performed as previously described (Desmaris et al. 2018; Theil 2005). After rehydration, a step of antigen retrieval is added for BrdU immunostaining as described below. The following primary antibodies were used: rabbit anti-Dmrt5 (gift from M. Li lab; 1:2000; (De Clercq et al. 2018)); rabbit anti-Nurr1 (Santa Cruz; sc-376 984; 1:500); goat anti-Nurr1 (R&D Systems; AF2156; 1:100); mouse anti-GFP (Molecular Probes; A11120; 1:500); rabbit anti-MAP2 (Sigma-Aldrich; M3096; 1:200); rabbit anti-Calretinin (Chemicon-Millipore; AB5054; 1:1000); rabbit anti-Tbr1 (Abcam; ab31940; 1:100); mouse anti-BrdU (Sigma-Aldrich; B2531; 1:1000); rat anti-Ctip2 (Abcam; ab18465; 1:500); rabbit anti-Tbr2 (Abcam; ab23345; 1:500); mouse anti-BLBP (Chemicon-Millipore; ABN14; 1:500); mouse anti-RC2 (DSHB; AB_531887; 1:500); rabbit anti-GFAP (Dako; 20 334; 1:500); rabbit anti-γ-tubulin (Abcam; ab11317; 1:400); rabbit anti-Ki67 (Novacastra; NCL-Ki67p; 1:500); mouse anti-Pax6 (DSHB; AB_528427; 1:50); rabbit anti-Hippocalcin (Abcam; ab24560; 1:2500) and rabbit anti-Pcp4 (Proteintech; 19 230; 1:250); and mouse anti-Reelin (Millipore; MAB5364; 1:1000). Secondary antibodies were goat anti-rabbit or goat anti-mouse Alexa 488 (green) or Alexa 594 (red) (Invitrogen; A11008 and A11005; 1:400), goat anti-rat Alexa 594 (red) (Invitrogen; A11007; 1: 400), donkey anti-goat 647(Abcam; Ab150131 1:100), and Cy2- and Cy3-conjugated to secondary antibodies anti-mouse and rabbit (Pc4, Reelin and Hippocalcin staining) (Dianova). Sections were counterstained with Hoechst (62 249; ThermoFischer Scientific). Images of immunofluorescence were acquired with a Zeiss LSM70 confocal microscope using Zeiss Zenblack© software (Zeiss). For Tile scan imaging, acquisitions were performed with a 10% overlap of fields and images were reconstructed using ZenBlack© software. Images were processed using Image J software and compiled using Adobe Photoshop CS3©.

All experiments have been done on brains from at least two animals from two different litters. The number of animals used for each ISH and IF experiment in the different figures is indicated in Table 1.

**Table 1:** Summary of brain numbers (*n*) used for each figure and application

Figure and application		Genotype	Age	Preparation	Number
Fig. 1	Immunofluorescence	*Wild type*	E11.5, E12.5, E13.5, E14.5, E15.5, E16.5	Immersion fixed (PFA) and frozen	*n* = 3, 3, 2, 3, 3, 2
		*Dmrt5^−/−^*			*n* = 3, 3, 2, 3, 3, 2
Fig. 2	Immunofluorescence	*Lpar1-eGFP*	E14.5	Fresh frozen	*n* = 3 (from 3 litters)
		*Wild type*	E16.5	Immersion fixed (PFA) and frozen	*n* = 2 (from 2 litters)
Fig. 3	ISH	*Wild type*	E12.5; E18.5	Immersion fixed (PFA) and frozen	*n* = 3
		*Dmrt5^−/−^*			*n* = 3
	Immunofluorescence	*Wild type*	E11.5, E12.5, E13.5, E15.5		*n* = 3
		*Dmrt5^−/−^*			*n* = 3
Fig. 4	ISH	*Wild type*	E15.5; E18.5	Immersion fixed (PFA) and frozen	*n* = 3, 3
		*Dmrt5^−/−^*			*n* = 3, 3
	Immunofluorescence	*Wild type*	E15.5		*n* = 2
		*Dmrt5^−/−^*			*n* = 2
Fig. 5	Immunofluorescence	*Wild type*	E15.5, E18.5	Immersion fixed (PFA) and frozen	*n* = 3,3
		*Dmrt5^−/−^*			*n* = 3,3
		*Dmrt5^lox/lox^; Emx1^CRE^*			*n* = 3,3
		*Dmrt5^lox/lox^; Nestin^CRE^*			*n* = 2,3
Fig. 6	Immunofluorescence	*Wild type*	E18.5 (BrdUE11.5; BrdUE12.5, BrdUE15.5)	Immersion fixed (PFA) and frozen	*n* = 3,3,2
		*Dmrt5^−/−^*			*n* = 3,3,2
Fig. 7	Immunofluorescence	*Wild type*	E18.5 (BrdUE11.5; BrdUE12.5, BrdUE15.5)	Immersion fixed (PFA) and frozen	*n* = 3,3,3
		*Dmrt5^−/−^*			*n* = 3,3,3
		*Wild type*	E18.5	Immersion fixed (PFA) and frozen	*n* = 4
		*Dmrt5^−/−^*			*n* = 4
Fig. 8	Immunofluorescence	*Wild type*	E15.5, E18.5		*n* = 2
		*Dmrt5^−/−^*			*n* = 2
		*Wild type*	E18.5	Dissociated culture fixed (PFA)	*n* = 3 (from 3 litters)
		*Dmrt5^−/−^*			*n* = 3 (from 3 litters)
Fig. S1	Immunofluorescence	*Wild type*	E10.5, E11.5, E12.5	Immersion fixed (PFA) and frozen	*n* = 3 (from 3 litters)
		*Dmrt5^−/−^*			
Fig. S2	Immunofluorescence	*Wild type*	E11.5, E12.5, E13.5, E14.5; E15.5	Immersion fixed (PFA) and frozen	*n* = 2, 3, 3, 3, 2
		*Dmrt5^−/−^*			*n* = 3, 3, 3, 3, 2
Fig. S3	Immunofluorescence	*Wild type*	E12.5	Immersion fixed (PFA) and frozen	*n* = 3
		*Dmrt5^−/−^*			*n* = 3
	Immunohistochemistry	*Wild type*			*n* = 3
		*Dmrt5^−/−^*			*n* = 3
Fig. S4	Immunofluorescence	*Wild type*	E18.5	Immersion fixed (PFA) and frozen	*n* = 3
		*Dmrt5^−/−^*			*n* = 3
		*Dmrt5^lox/lox^; Emx1^CRE^*			*n* = 3
		*Dmrt5^lox/lox^; Nestin^CRE^*			*n* = 2
Fig. S5	Immunofluorescence	*Wild type*	E18.5	Immersion fixed (PFA) and frozen	*n* = 2
		*Dmrt3^−/−;^Dmrt5^−/−^*			*n* = 2
	ISH	*Wild type*			*n* = 2
		*Dmrt3^−/−;^Dmrt5^−/−^*			*n* = 2

### Cell Proliferation, Cell Cycle Dynamics, and Birthdate Studies

For birth dating studies, timed-pregnant mice were injected intraperitoneally at several stages of pregnancy with a single pulse of 5′-bromo-2′-deoxyuridine (BrdU) (100 μg BrdU/g of body weight). Subsequently, distribution of BrdU-positive cells was determined at E18.5. Counted BrdU-positive cells are circular objects with ~50% of the structure filled. In order to investigate cell proliferation, BrdU was delivered 24 h before cervical dislocation. Brains were fixed as described before. Sections were subsequently prepared (thickness of 25 μm). Samples were first incubated in 2 N HCl for 60 min, followed by a 5 min treatment in 0.1 M Borate buffer (pH 8.5) to neutralize residual acid. Specimens were then immunostained with mouse anti-BrdU (Sigma-Aldrich; B2531; 1:1000) and specific markers (Nurr1, Ctip2, and Ki67) followed by secondary antibody coupled to anti-mouse AlexaFluor-488 (Invitrogen; A11008; 1:400).

For quantification of cells expressing Pax6 and Tbr2, rectangular fields of ~ 250 μm of width were selected from the pallium-subpallium boundary to the lateral cortex in rostral, medial, and caudal regions.

For Pax6 and Tbr2 nuclei counting and proliferative index experiments, a homemade automated macro was developed on ImageJ software (“Nuclei counting strategy with Fiji” (Desmaris et al. 2018)). Briefly, the background of images was reduced using a “rolling ball radius” function, and nuclei were segmented by fluorescence intensity using an automated threshold. Nuclei segmented from both “green” and “red” channels were counted automatically through a size selection and nuclei present in both channels were considered as colocalizing. Brightness and contrast adjustments and image processing were done using ImageJ and Adobe Photoshop CS3® software.

All quantified data are expressed as mean values ± standard deviation (SD). Significance tests were performed using an unpaired Student’s *t*-test; *P*-values less than 0.05 were regarded as statistically significant or using Mann-Whitney test when distribution does not pass the D’Agostino-Pearson normality test.

### Dissociated Cortical Neuron Cultures from Embryonic Mouse Brains

Embryos were dissected out from the uterus, and the brains were removed and placed in a sterile petri-dish with 5-mL cold L-15 medium (ThermoFisher; 11 415 114) on ice. Genotyping was performed as previously described (Saulnier et al. 2013) to confirm phenotypic selection of *WT* and *Dmrt5*^−/−^ brains. Brains were glued onto the support block of the vibroslicer (Leica VT1200S©) with the caudal side up and ventral part facing the agar block. Coronal sections were obtained (500-μm thick), and 3–4 slices containing the cortex were collected for further use. Neocortex was dissected and cut into small pieces from brain sections. Cortical tissue pieces were transferred to Eppendorf tubes for dissociation with 0.05% Trypsin (Invitrogen; 17 075 029) followed by treatment with trypsin inhibitor solution (Sigma; D4513) as described previously (Muralidharan et al. 2017; Young et al. 2017). Cells were resuspended in Neurobasal medium containing B-27 supplement (Invitrogen; 10 889 038) containing 10% fetal bovine serum (Fisher scientific; 11 573 397). The optimal cell density in the culture was determined to 15 000cells/cm^2^, and lysates were diluted to finally spread 200 μL of cell suspension per coverslip. Coverslips were previously coated with poly-D-lysine/laminin (Sigma, P27280) and placed in a 12-well plate. They were then placed in an incubator at 37 °C in a 5% CO_2_ atmosphere. After 1 h, cells are attached to the coverslips and the 12-well plates were filled with 2 mL of medium. After 2 days *in vitro* at 37 °C in a 5% CO_2_ atmosphere, the cells were fixed with PFA 4% for 30 min at 4 °C, rinsed with cold PBS, and processed for immunofluorescence as previously described in Muralidharan et al. 2017.

## Results

### Transient Disruption of the Mitotic Dynamics in *Dmrt5*^−/−^ Neocortex

Previous work revealed that *Dmrt5* is required for cortical growth and patterning (De Clercq et al. 2018; Konno et al. 2012; Saulnier et al. 2013; Young et al. 2017). To better characterize the mechanisms of *Dmrt5* action during corticogenesis, we compared the plane of apical progenitor cell divisions and cell cycle exit rates of *Dmrt5*^−/−^ and *WT* embryos (Figs S1 and S2). The proportion of oblique division is increased from metaphase to ana-telophase in E10.5 but not in E11.5 or E12.5 *Dmrt5*^−/−^ embryos (Fig. S1). Moreover, we observed that the cell cycle exit rate is also transiently perturbed in *Dmrt5*^−/−^ cortices at E12.5, but the proliferation of cortical progenitors remained comparable to *WT* at later ages (E13.5, E14.5, and E15.5; Fig. S2). This transient change in mitotic dynamics may partially contribute to the drastic reduction and the poor differentiation of the cortical wall of E18.5 *Dmrt5*^−/−^ embryos, but it is unlikely that it fully explains the severe phenotype.

### Loss of *Dmrt5* Affects the Ratio of Apical to Basal Progenitors in the Lateral Cortex

**Figure 1:**
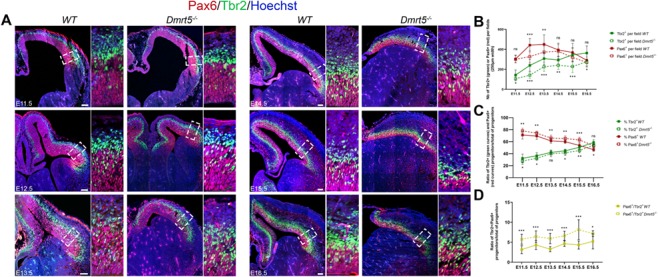
Loss of Dmrt5 affects the number of apical and basal progenitors in the developing lateral cortex. (*A*) Coronal sections of brains from E11.5, E12.5, E13.5, E14.5, E15.5 and E16.5 *WT* and *Dmrt5^−/−^* embryos were immunostained with Pax6 (red) and Tbr2 (green) antibodies and counterstained with Hoechst (blue). Enlarged images from the areas outlined with dashed boxes depict examples of counting areas. (*B*) Graph showing the total number of Pax6^+^ (red) or Tbr2^+^ (green) progenitors per section at each stage of development of *WT* (solid lines) and *Dmrt5^−/−^* (dotted lines) embryos. The number of Tbr2^+^ and Pax6^+^ cells is reduced in *Dmrt5*^−/−^ compared with *WT* embryos. (*C*) Graph showing the ratio of Pax6^+^ progenitors (red) or Tbr2^+^ progenitors (green) to the total number of progenitors in *WT* (solid lines) and *Dmrt5^−/−^* (dotted lines) embryos at each indicated stage. This ratio is decreased in the case Tbr2 and increased for Pax6 in *Dmrt5*^−/−^compared with the *WT* embryos. (*D*) Graph showing the ratio of double-labeled Pax6^+^Tbr2^+^ cells compared with the total of progenitors at each indicated stage in *WT* (solid line) and *Dmrt5*^−/−^ (dotted line) embryos. The transitioning cell population is larger in *Dmrt5*^−/−^ compared with *WT.* Scale bars in *A* represent 100 μm.

We examined the ratio of apical and basal neural progenitor (NP) populations in the cortex of *Dmrt5*^−/−^ and *WT* embryos. We used immunohistochemistry for Pax6 (paired-box protein 6) and Tbr2 (T-box transcription factor 2) to identify the nuclei of apical (including neuroepithelial and radial glia) or intermediate/basal progenitors, respectively (Englund et al. 2005). In *Dmrt5*^−/−^ embryos, we previously demonstrated that the Tbr2^+^ cell population is transiently increased in the medial part of the telencephalic vesicles where the *Dmrt5* expression gradient would normally be at its highest (Saulnier et al. 2013). Since Tbr2 expression has a lateral (high) to medial (low) gradient, we focused our analysis on lateral cortex above the pallial-subpallial boundary (Fig. 1*A*, dotted box). We observed that the number of Tbr2^+^ progenitors was reduced in *Dmrt5*^−/−^ compared with *WT* at all ages studied (Fig. 1*B*, E11.5: *P* = 0.04; E12.5: *P* = 7.63*E* − 06; E13.5: *P* = 1.8*E* − 04; E14.5: *P* = 0.008; E15.5: *P* = 8.8*E* − 04; and E16.5: *P* = 0.038). These results demonstrate that Tbr2 expression is disturbed in *Dmrt5*^−/−^ brains. Additionally, the number of Pax6^+^ cells per region of interest was also significantly decreased between E12.5 and E13.5, during the peak of layer VI neurogenesis (E12.5: *P* = 6.51*E* − 05; E13.5: *P* = 2.69*E* − 08). The timing of the transient depletion of Pax6^+^ progenitors correlates with the previously described burst of neuron production in *Dmrt5*^−/−^ embryos (Saulnier et al. 2013). Our analysis of the ratio of Tbr2^+^ or Pax6^+^ cells to the total number of progenitors revealed that this ratio was lower for Tbr2 and higher for Pax6 in *Dmrt5*^−/−^ brains compared with WT (Fig. 1*C*). These results suggest that the loss of *Dmrt5* affects the ratio of apical to basal progenitors in the lateral cortex, where apical progenitors could generate less intermediate progenitor cells (IPCs) and/or their differentiation is slowed down.

To examine this possibility, we studied the double labeled Pax6^+^Tbr2^+^ cells that correspond most probably to cells transitioning from radial glial progenitor cells (RGCs) to IPCs (Fig. 1*D*). We found that the ratio of Pax6^+^Tbr2^+^ progenitors to total the number of progenitors was higher at all developmental time points examined in *Dmrt5*^−/−^ compared with *WT* embryos (Fig. 1*D*, E11.5: *P* = 1.05*E* − 05; E12.5: *P* = 1.1*E* − 04; E13.5: *P* = 2.69*E* − 08; E14.5: *P* = 1.1*E* − 04; E15.5: *P* = 2.14*E* − 04; and E16.5: *P* = 0.042). Assuming that the probability that a cell which is transiting between a radial progenitor to an intermediate progenitor state will express both Pax6 and Tbr2 is the same in the mutant versus the WT, these results suggest that indirect neurogenesis through IPCs is more prevalent compared with direct neurogenesis from RGCs in the lateral cortex of *Dmrt5^−/−^* embryos.

### A Subset of SPns Show Dmrt5 Immunoreactivity

**Figure 2:**
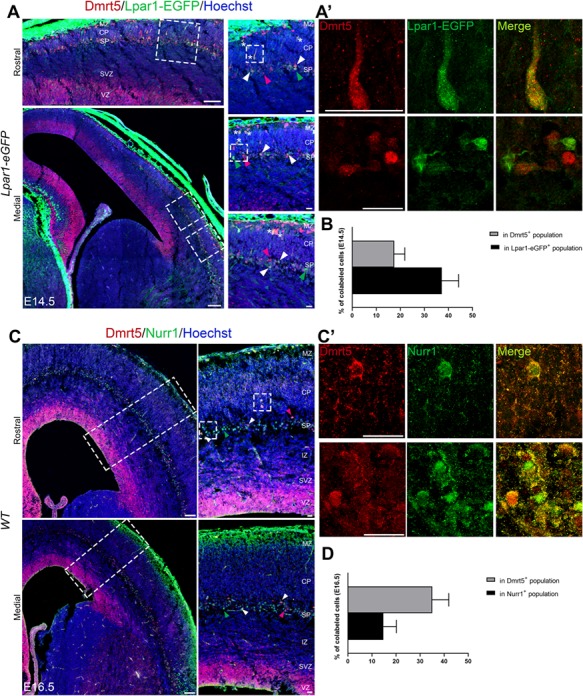
Dmrt5 is co-expressed with a subset of SP markers. (*A*, *B*) Coronal sections of the brain of E14.5 *Lpar1*-*eGFP* embryos immunostained for Dmrt5 (*A*) and of E16.5 embryos immunostained for Dmrt5 and Nurr1 (*C*). Areas outlined with dashed boxes are shown at higher magnification on the right (*A’* and *C’*). White arrowheads in the insets indicate examples of co-labeled cells. Green and red arrowheads indicate Dmrt5^−^Lpar1-eGFP^+^ and Dmrt5^+^Lpar1-eGFP^−^ cells, respectively, in *A*, and Dmrt5^−^Nurr1^+^ and Dmrt5^+^Nurr1^−^ cells in *C*. Asterisks in right panels of *A* indicate Lpar1-GFP^+^Dmrt5^+^ co-labeled cells with neuronal morphology in CP. The proportion of co-labeled cells in the Lpar1-eGFP^+^ (37.2% ± 7.0) or Dmrt5^+^ populations (17.5% ± 4.4) at E14.5 (*B*; *n* = 12 sections from three brains) and in Nurr1^+^ (14.7% ± 5.6) or Dmrt5^+^ populations (35.1% ± 6.8) at E16.5 (*D*; *n* = 6 sections from two brains) are shown in the graphs. Data given are mean ± SD. Scale bars represent 100 μm in *A* and *C* and 25 μm in high-magnification boxes (*A’* and *C’*).

IPCs contribute to all layers of the cortex including SP, and disruption in Tbr2 expression leads to defects in neuron specification (Englund et al. 2005; Mihalas et al. 2016; Vasistha et al. 2015). In *Dmrt5*^−/−^ brains, the reduction of cortical thickness is associated with an absence of the SP (Saulnier et al. 2013). To better understand the possible origin of the cortical defects in *Dmrt5*^−/−^ embryos, we studied *Dmrt5* expression during development. As previously reported by Saulnier and colleagues, we observed strong nuclear Dmrt5 immunoreactivity in cortical progenitors and in Cajal-Retzius cells in the MZ (Saulnier et al. 2013). In addition to these two areas, we detected Dmrt5 immunoreactivity in a band of cells between the germinative zone and CP corresponding to the SP layer (Fig. 2). Moreover, we also observed Dmrt5 immunoreactivity in some scattered elongated neuron-shaped cells within the CP. Such cells are marked by asterisks in Figure 2*A*, and a high-magnification view of them is shown (Fig. 2*A*, top right panels).

To determine the location of these Dmrt5^+^ cells in relation to SPns, we used the Lpar1-eGFP mouse line (Hoerder-Suabedissen et al. 2013; Hoerder-Suabedissen and Molnár 2015) where GFP-expression is present both in SPn and GABAergic interneurons in layers V and VIa (Hoerder-Suabedissen and Molnár 2015; Marques-Smith et al. 2016). Dmrt5 immunoreactivity was co-expressed with eGFP in Lpar1-eGFP^+^ in some SP cells (Fig. 2*A*, bottom right panels). The proportion of co-staining within the Lpar1-eGFP^+^ population is higher (37%) than within the Dmrt5^+^ population (17.5%) (Fig. 2*A*). At E16.5, around 35% of Dmrt5^+^ cells were also Nurr1^+^. Nurr1 is an orphan nuclear receptor (Nr4a2) and a typical marker of SP cells (Hoerder-Suabedissen et al. 2009; Fig. 2*B*). Thus, a subset of SPn is Dmrt5 immunoreactive. Based on the partial overlap of Dmrt5 with Nurr1 immunoreactivity and with Lpar1-eGFP expression, several subpopulations of SPn expressing Dmrt5 can be defined (Dmrt5^+^Lpar1-eGFP^−^; Dmrt5^+^Nurr1^−^; Dmrt5^+^Nurr1^+^; and Dmrt5^+^Lpar1-eGFP^+^). The fact that some of the elongated Dmrt5^+^ neurons in the CP are also Lpar1-eGFP^+^suggests that they may be SPns in migration into and within the CP.

Neurons of the SP layer have multiple origins. Some glutamatergic SPns are born in the pallial *Emx1* cortical territory (Shinozaki et al. 2002; Yoshida et al. 1997). Other glutamatergic SP cells are generated in the rostral medial telencephalic wall (RMTW) and migrate tangentially to SP (García-Moreno et al. 2008; Pedraza et al. 2014) and some GABAergic SPns that originate from the subpallial ganglionic eminence displayed long-range axonal projections (Lavdas et al. 1999; Le Magueresse and Monyer 2013; Boon et al. 2019). With the exception of the subpallial ganglionic eminence, *Dmrt5* is expressed in the germinal zone of the cerebral cortex and RMTW, similarly to *Emx1*, which suggests that Dmrt5 protein expression might be associated with the generation and maintenance of some glutamatergic SPns. Although the *Dmrt5* expression is lower in rostral telencephalon, we also observed Dmrt5 immunoreactivity in the medial part of the rostral telencephalic wall. Immunohistochemistry for Dmrt5 on Lpar1-eGFP sections revealed a co-labeling of Dmrt5 immunoreactivity in the eGFP expressing RMTW (data not shown). Thus, SPns originating from the cerebral cortex or from the RMTW could both contribute to Dmrt5^+^Lpar1-eGFP^+^ or Dmrt5^+^Nurr1^+^ SP populations that migrate radially or tangentially, respectively, to the cortex.

### *Dmrt5* and *Dmrt3* Are Required for Early SP Layer Formation and Their Loss Leads to a Disorganized CP

**Figure 3:**
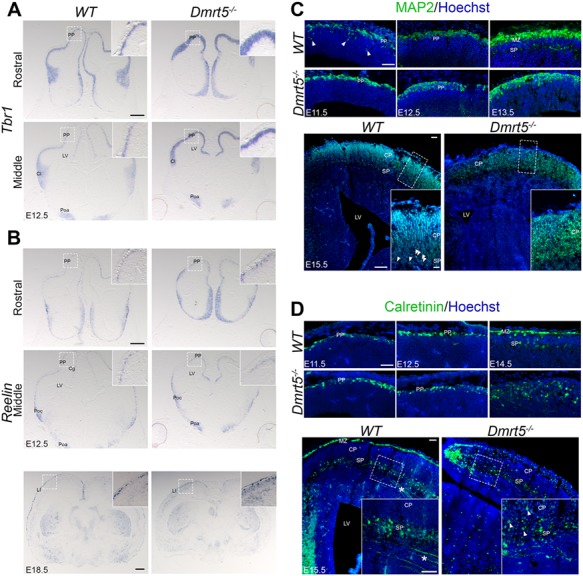
Early SP development and subsequent cortical neuron migration are affected in *Dmrt5* mutants. (*A*, *B*) ISH for (*A*) *Tbr1* and (*B*) *Reelin* in coronal sections from *WT* and *Dmrt5*^−/−^ embryos at E12.5 (and E18.5 for *Reelin*). *Tbr1* and *Reelin* have a comparable distribution in PP, Cl, and Poc; Poa and Cg in *WT* and *Dmrt5*^−/−^ embryos at E12.5. Note that *Tbr1* is overexpressed in *Dmrt5*^−/−^ embryos (*A*). Note that while Cajal-Retzius and PP neurons are detectable in the cortex of both *WT* and *Dmrt5*^−/−^ embryos at E12.5, *Reelin* expression is decreased at E18.5 in *Dmrt5*^−/−^ compared with WT brains and fewer *Reelin*^+^ cells are located in the MZ (*B*). In each panel, a high-magnification image of the boxed area is shown. (*C*, *D*) Coronal sections of developing brains from *WT* and *Dmrt5*^−/−^ embryos from E11.5 to E15.5 were immunostained for MAP2 (*C*) and Calretinin (*D*) and counterstained with Hoechst (blue). Views of the lateral cortex at various stages are shown, with high magnification of the boxed area in insets. At E13.5–E15.5, MAP2 is detected in CP and SPn (white arrowheads in high-magnification box of E15.5 WT) in the cortex of WT embryos, whereas no cytoarchitectonically distinct SP can be detected in the cortex of *Dmrt5*^−/−^ embryos, the majority of the CP neurons showing orientation defects. In *WT*, Calretinin is expressed in the SP layer and in the MZ. Some of the earliest corticofugal projections are also immunoreactive and extend through the intermediate zone towards the internal capsule in *WT* and *Dmrt5*^−/−^ (white asterisk in lower left panel at *D*). Note that the disorganized band of calretinin positive cells in the SP region of the cortex of *Dmrt5*^−/−^embryos, and the presence of ectopic Calretinin^+^ cells within the CP with abnormal shape (white arrowheads). Clumpings of Calretinin^+^ cells are observed at the edge of the medial cortex close to Hpc in *WT* and *Dmrt5*^−/−^ embryonic brains. Scale bars represent 50 μm for immunofluorescence images and 500 μm for ISH images. Cg: cingulate; CP: cortical plate; LV: lateral ventricle; Poa: preoptic area; Poc: prospective olfactory cortex.

Based on the data above suggesting that Dmrt5 is expressed in the SP, and the defective SP development in *Dmrt5*^−/−^ embryos (Saulnier et al. 2013), we considered the role of *Dmrt5* in splitting the PP. The PP is the first postmitotic cell layer of the cortex that later gives rise to MZ and SP (Hevner et al. 2003; Kwan 2013; Marin-Padilla 1971; Stewart and Pearlman 1987). In *Dmrt5*^−/−^ and *WT* embryos, we examined the expression of PP and early SP markers by *ISH* or immunostaining at E12.5 (Figs 3*B* and S3). Among them, *Tbr1* was expressed in the PP and later in SP and layer VI. A thicker *Tbr1*^+^ band was observed in *Dmrt5*^−/−^ compared with WT (Fig. 3*A*). We also studied *Reelin* expression in Cajal-Retzius cells. These cells migrate tangentially from peripheral regions into the PP of the developing cortex and integrate into the MZ/layer I (LI) of the mature cortex (Bielle et al. 2005; Martinez-Cerdeno and Noctor 2014). In accordance with previous observations (Saulnier et al. 2013), we observed fewer *Reelin*^+^ cells in *Dmrt5*^−/−^ brains than in *WT* controls. The expression of the SP markers Pcp4 (Purkinje cell protein 4) (Arlotta et al. 2005; Renelt et al. 2014) and Hippocalcin (Osheroff and Hatten 2009) were also examined. The staining of these two markers was also strongly reduced or absent in the mutant dorsal telencephalon (Fig. S3).

While MAP2 is a general neuronal marker, Calretinin is restricted to SPn and MZ/LI and some CP neurons (Espinosa 2009; Hevner et al. 2001; Ina et al. 2007; Kwan 2013; Theil 2005). We used Calretinin and MAP2 immunohistochemistry to determine whether the formation of a layered MZ, SP, and CP is altered in *Dmrt5*^−/−^ embryos from E11.5 to E15.5. From E11.5 to E12.5, the distribution of MAP2 and Calretinin immunoreactive neurons appeared comparable in the PP between *WT* and *Dmrt5*^−/−^ brains (Fig. 3*C*,*D*). However, at E13.5, MAP2 staining in the SP layer was absent in *Dmrt5*^−/−^ cortex (Fig. 3*C*). At E11.5 and E12.5, the Calretinin immunoreactive neurons are in the PP in both WT and *Dmrt5*^−/−^ brains. However, by E14.5, Calretinin immunoreactive cells are split by the forming CP and they are localized to the MZ and the SP in *WT*. In *Dmrt5*^−/−^ brains, the Calretinin immunoreactive cells do not split and they are scattered within a large band of disorganized cells (Fig. 3*D*). By E15.5, the majority of MAP2 mature neurons detected in the *Dmrt5*^−/−^ CP exhibited defects in orientation within the CP (Fig. 3*C*, high-magnification boxes). Calretinin immunoreactive cells were much sparser in the MZ, and a disorganized band of Calretinin immunoreactive cells was detected in the SP region of *Dmrt5*^−/−^ embryos (Fig. 3*D*). Large aggregate of Calretinin^+^ cells were often found at the medial edge of cortex as was previously described (Saulnier et al. 2013). We also detected ectopic Calretinin^+^ cells that do not exhibit processes in *Dmrt5*^−/−^ embryos (Fig. 3*D*, white arrowheads).

**Figure 4:**
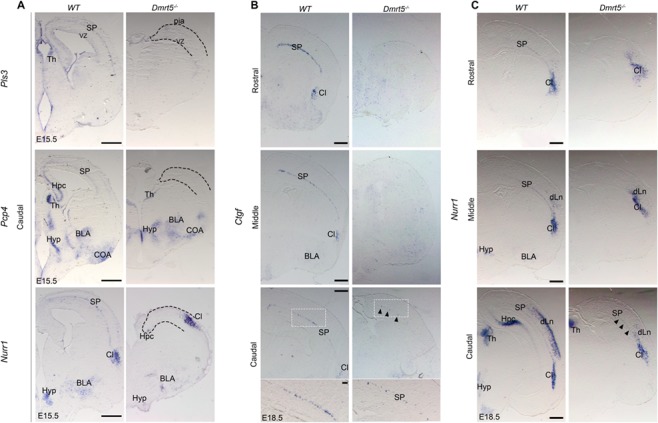
SP defects are more severe in the rostral than in the caudal part of the cortex of *Dmrt5*^−/−^ embryos. (*A*–*C*) ISH for *Pls3*, *Pcp4*, and *Nurr1* in coronal sections from *WT* and *Dmrt5*^−/−^ embryos at E15.5 (*A*) and for *Ctgf* and *Nurr1* at E18.5 (*B* and *C*). Expression of the SP markers *Pcp4*, *Pls3*, and *Nurr1* is strongly reduced in the neocortex and Hpc at E15.5 in *Dmrt5^−/−^.* At E18.5, *Ctgf* and *Nurr1* expressions were however detected in the SP within the caudal part of the cortex (black arrowheads). Higher magnification of the boxed area is shown for *Ctgf* staining in caudal sections. dLn: deep layers neurons; EN: endopiriform nucleus; VZ: ventricular zone. Scale bar represents 500 μm for all low power images in *A*–C and 100 μm for lower panels in *B*.

**Figure 5:**
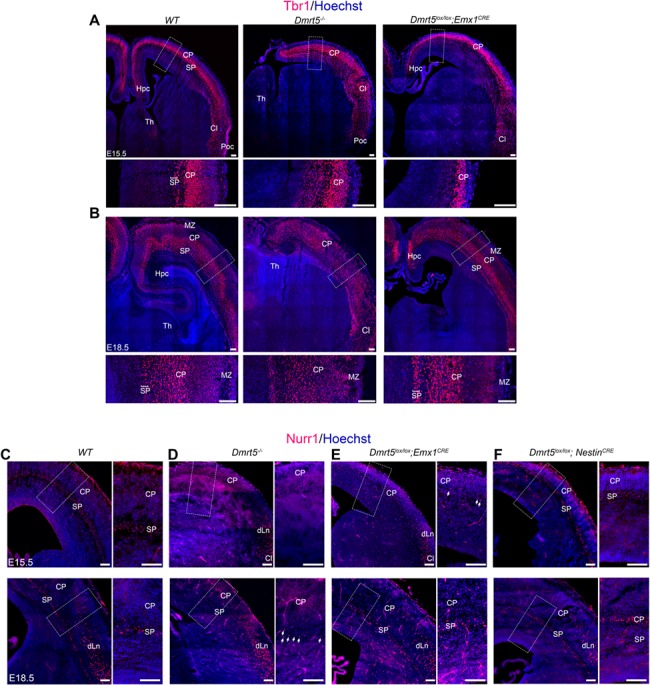
Dmrt5 function is explored in various transgenic mouse models (*WT*, *Dmrt5*^−/−^, *Dmrt5*^*lox*/lox^;*Emx1*^*CRE*+^ and *Dmrt5*^*lox*/lox^;*Nestin*^*CRE*+^) with different time-points of Cre-recombination. (*A*, *B*) Coronal sections from E15.5 (*A*) and E18.5 (*B*) *WT* and *Dmrt5*^−/−^ brains that were immunostained for Tbr1 (red) and counterstained with Hoechst (blue). At E15.5, Tbr1-immunoreactive cells were present in CP, and Cl in the *Dmrt5*^−/−^ and *Dmrt5^lox/lox^;Emx1^Cre^* similarly to *WT*. The enlarged images from the regions indicated with boxes demonstrate that the SP is present in *WT*, but absent in *Dmrt5*^−/−^ and *Dmrt5^lox/lox^;Emx1^Cre^*. Moreover, the CP is reduced in thickness in *Dmrt5*^−/−^ and in *Dmrt5^lox/lox^;Emx1^Cre^*. At E18.5, the SP layer is present in *Dmrt5^lox/lox^;Emx1^Cre^* brains. In *Dmrt5*^−/−^, the separation between the CP and SP is not evident with Tbr1 immunohistochemical or Hoechst stainings. (*C*–*F*) Coronal sections of developing brains from *WT* (*C*), *Dmrt5*^−/−^ (*D*), *Dmrt5*^*lox*/lox^; *Emx1*^*CRE*+^ (*E*), and *Dmrt5*^*lox*/lox^;*Nestin*^*CRE*+^ (*F*) immunostained for Nurr1 at E15.5 (upper row) and E18.5 (lower row). Enlarged images of the regions of dorsal cortex indicated by the boxes are presented for each brain. SP was not visible at E15.5, but sparse Nurr1^+^ SP cells are detected at E18.5 in *Dmrt5*^−/−^ brains (white arrows in the high-power image of D). Nurr1 expression is absent at E15.5 in *Dmrt5*^*lox*/lox^;*Emx1*^*CRE*+^ embryonic brain even though the SP layer was later comparable to the *WT* at E18.5. Nurr1 expression is not affected in SP in *Dmrt5*^*lox*/lox^;*Nestin*^*CRE*+^ embryonic brains. Nurr1 immunoreactivity is detected in dLns in the lateral cortex and in the Cl of *WT*, *Dmrt5*^−/−,^*Dmrt5*^*lox*/lox^; *Emx1*^*CRE*+^ and *Dmrt5*^*lox*/lox^;*Nestin*^*CRE*+^. Scale bars represent 100 μm for all images.

To further characterize SP defects in *Dmrt5*^−/−^ brains, we analyzed the expression of different SP markers at later stages. At E15.5, *Pls3* (*Plastin 3*), which is expressed in a subset of SPn and lower CP neurons in *WT* brains (Oeschger et al. 2012), was not detectable anywhere in the brain of *Dmrt5*^−/−^ embryos (Fig. 4*A*). *Pcp4* and *Nurr1* were both also undetectable in the SP of mutant brains, while they displayed similar extracortical expression in the hypothalamus, thalamus, basolateral amygdala anterior (BLA), cortical amygdaloid nucleus, and claustrum (Cl) (Fig. 4*A*) (Arimatsu et al. 2003). We also examined Tbr1 immunoreactivity that is normally detected in CP and SP in *WT* brains, but a Tbr1 immunoreactive SP layer was not detectable below the CP at E15.5 in *Dmrt5*^−/−^ brains (Fig. 5*A*). At E18.5, staining for *Nurr1* and *Ctgf* (Connective tissue growth factor) that is selectively expressed in most late born SPn (Hoerder-Suabedissen et al. 2009; Wang et al. 2010) were reduced in *Dmrt5*^−/−^ embryos, and the SP defects appear more severe rostrally than caudally (Fig. 4*B*,*C*; black arrowheads). *Nurr1* expression was also reduced in dLn (Fig. 4; scattered cells and Fig. S4) as in the anlage of the hippocampus (Hpc) (Figs 4 and S4, black arrow) but appears unaffected in the Cl. These results are consistent with previous findings indicating that the loss of Dmrt5 leads to the disorganization of cortical layers, precocious cortical neurogenesis, and defective SP development (Saulnier et al. 2013; Young et al. 2017). They further suggest that Dmrt5 may be required for SP fate specification.

We previously showed that Dmrt3 and Dmrt5 may act redundantly in different aspects of cortical development and may compensate for the loss of each other (De Clercq et al. 2018; Desmaris et al. 2018). We therefore analyzed the expression of SP markers in *Dmrt3*^−/−^ and *Dmrt3*^−/−^;*Dmrt5*^−/−^ embryos. We visualized SPn through their Nurr1 immunoreactivity at E18.5 in *Dmrt3*^−/−^ embryos (Fig. S4). The SP defects in *Dmrt5*^−/−^ appeared more severe rostrally than caudally (Fig. S4, black arrowheads). In *Dmrt5^−/−^;Dmrt3^−/−^* double mutant embryos, where the cortex is nearly absent (Desmaris et al. 2018), Nurr1, Tbr1, and *Gap43* expressions in the dorsal brain were undetectable (Fig. S5). By contrast, the Nurr1 immunoreactive claustral neurons were present in the single and double mutants (Cl; Figs S4 and S5). These results indicate that *Dmrt3* also contributes to the regulation of SP formation.

### Restricted Developmental Period for *Dmrt5* Action in SP Formation

*Dmrt5* is strongly expressed in early dorsal telencephalic progenitors and its expression declines with time during corticogenesis. The exact timeframe for Dmrt5 expression within cortical progenitors required for SP formation is not known. Therefore, we studied SP development in *Dmrt5*^−/−^ null knock-out animals and two *Dmrt5* conditional knock-out mouse strains, *Dmrt5^Lox/Lox^;Emx1^Cre^*, and *Dmrt5^Lox/Lox^;Nestin^Cre^* mice. These two conditional knock out strains have different timing of efficient deletion for *Dmrt5* (De Clercq et al. 2018). *Dmrt5* was disrupted from E10.5 in cortical progenitors in *Dmrt5^Lox/Lox^;Emx1^Cre^* mice and 1 day later in *Dmrt5^Lox/Lox^;Nestin^Cre^* mice (De Clercq et al. 2018; Gorski et al. 2002; Tronche et al. 1999). We used Nurr1 immunohistochemistry to reveal SPns. In *Dmrt5^Lox/Lox^;Emx1^Cre^* mice (Fig. 5*C*–*F*), only few Nurr1^+^ SPns were detected (Fig. 5*E*, white arrows), whereas Nurr1+ cells were present in the Cl and dLn in these brains at E15.5 (Fig. 5*E*). Surprisingly, a near normal layer of Nurr1^+^ cells was visible in the SP of these *Dmrt5^Lox/Lox^;Emx1^Cre^* embryos by E18.5 suggesting that SP formation is only delayed in this conditional mutant (Fig. 5*E*). Other SP markers such as *Pcp4* and Tbr1 were also absent in the SP at E15.5, whereas their expression was detected at E18.5 (Fig. 5*A*,*B* and data not shown). In contrast, Nurr1 immunoreactive cells in the SP of the *Dmrt5^Lox/Lox^;Nestin^Cre^* brains was very similar to *WT* mice, both at E15.5 and E18.5 (Fig. 5*C*,*F*). Thus, while *Dmrt*5 ablation from E10.5 appears to slow down SP formation, its ablation from E11.5 has no effect.

We also examined the consequences of the overexpression of Dmrt5 in Nurr1^+^ SPn population with *Dmrt*^*Tg*/Tg^; *Emx1Cre* conditional transgenic mice. Nurr1 was expressed in the SP, albeit with a slightly weaker signal at E18.5 compared with controls (Fig. S4). Thus, while *Dmrt5* is not sufficient to specify a SP fate, it is required in early cortical progenitors for their formation.

### Switch in Nurr1 and Ctip2 Neurogenesis in *Dmrt5*^−/−^ Cortex

**Figure 6:**
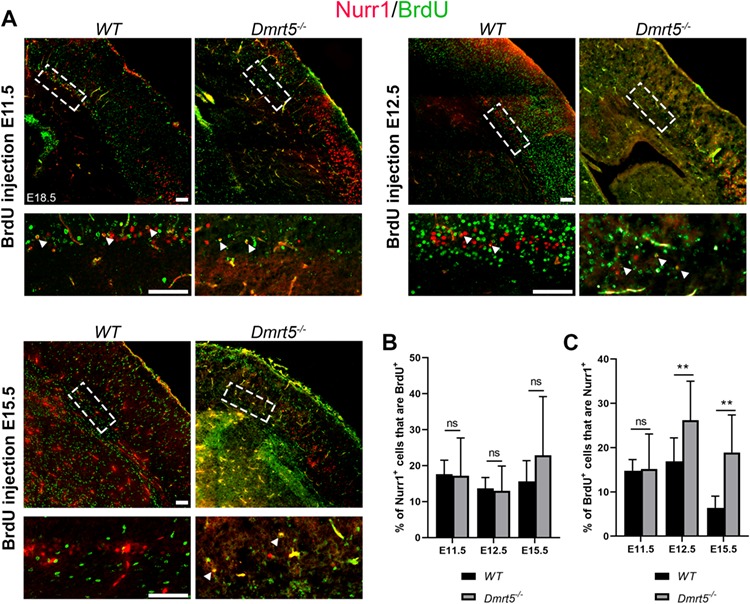
Nurr1^+^ SPns are born later in *Dmrt5*^−/−^. (*A*) To compare the birthdate of the Nurr1^+^ SPns in *WT* and *Dmrt5*^−/−^ cortex, a single pulse of BrdU was injected at E11.5; E12.5, or E15.5, and brains were collected and fixed at E18.5. Coronal sections of these birth dated brains from *WT* and *Dmrt5*^−/−^ were immunostained for Nurr1 and BrdU. The areas indicated with boxes in SP are also shown with higher magnification to show examples of co-labeled cells (indicated with arrowheads). (*B*) Quantification of Nurr1 immunoreactivity among the different BrdU^+^ SPn populations born at E11.5, E12.5, or E15.5 demonstrated that SPns acquire a Nurr1 identity in both *WT* and *Dmrt5*^−/−^ mice. To determine the proportion of Nurr1^+^ cells in the cohorts that are born at E11.5, E12.5, and E15.5 stages, we quantified the % of BrdU labeling in Nurr1 immunoreactive SPn (*n* = 3, 3, and 2 brains for the three stages; at least three sections of each brain) (*C*). There is a significant increase of the % of BrdU^+^ cells that are Nurr1^+^ for BrdU pulses given at E12.5 and E15.5 in the *Dmrt5*^−/−^ brains (26.2 ± 8.8% and 18.9 ± 8.5%) compared with *WT* (16.9 ± 5.3% and 6.4 ± 2.6%) suggesting a delay in SP generation in *Dmrt5*^−/−^ cortex, or a misspecification of later born neurons to an SPn phenotype. ^*^^*^P < 0.01 (unpaired Student’s *t-*test). Data are given as mean ± SD. Scale bars represent 100 μm for all images.

To explore the mechanism of SPn generation in *Dmrt5*^−/−^ embryos, we performed BrdU birthdating experiments. We gave a pulse of BrdU to pregnant females at either the peak of SP formation (E11.5 and E12.5) or later at E15.5 and determined the distribution of BrdU-labeled Nurr1 immunoreactive neurons at E18.5 (Fig. 6). We considered these cells as SPn because we only observed Nurr1 immunoreactivity and did not observe co-staining of Nurr1 and Ctip2 (Chicken ovalbumin upstream promoter transcription factor (Ctip2/Bcl11); a marker of layer V neurons, see below) in this area at E18.5. Our analysis revealed a drastic reduction in Nurr1^+^ cells in *Dmrt5*^−/−^ brains (35.7 ± 21.0 at E11.5; 23.5 ± 8,7 at E12.5; 14.1 ± 4.2 at E15.5 per field of view) compared with *WT* brains (119.1 ± 51.2 at E11.5; 119,3 ± 42,7 at E12.5; 72.75 ± 18.58 at E15.5 per field of view; ^*^^*^^*^P < 0.001). However, the cohorts of birth-dated cortical neurons at E11.5, E12.5, and E15.5 had similar proportion of Nurr1 immunoreactive neurons in *Dmrt5*^−/−^ and in *WT* brains (17.6% vs. 17.2% at E11.5; 13.7% vs. 13% at E12.5 and 15.6% vs. 22.9% at E15.5; ns *P* > 0.05) (Fig. 6*B*). While the majority of the SPns was born at E11.5 and E12.5 in *WT*, a higher proportion of Nurr1^+^ neurons was generated later at E12.5 in *Dmrt5*^−/−^ (26.2%) compared with *WT* embryos (16.9%; ^*^^*^P = 0.006), as well as at E15.5 (18.9% vs. 6.4%; ^*^^*^P = 0.003) (Fig. 6*C*). These data suggest a delay in the neurogenesis of SPn in the absence of *Dmrt5*, or misspecification of neurons.

**Figure 7:**
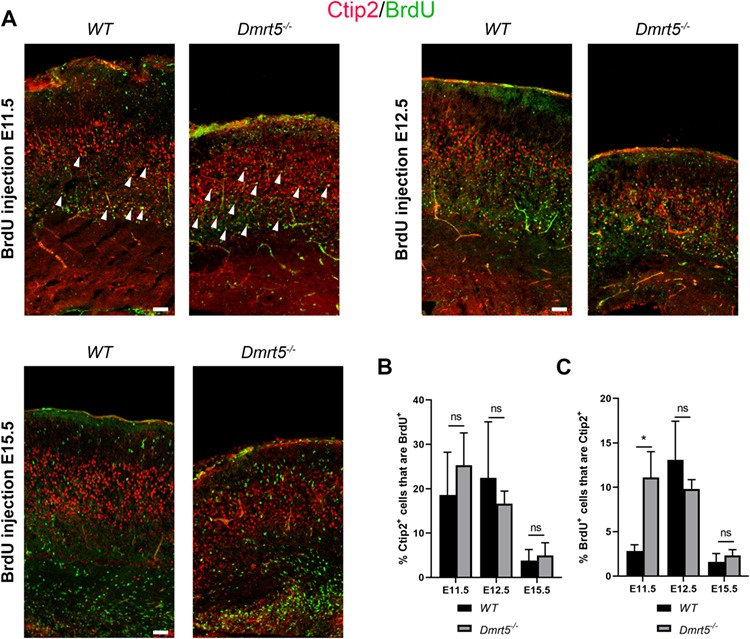
*Dmrt5* regulates the timing of the earliest Ctip2+ cortical neuron generation. (*A*) To compare the birthdate of Ctip2 immunoreactive cohorts of cortical neurons in *WT* and *Dmrt5*^−/−^, BrdU was injected at E11.5, E12.5, or E15.5, and brains were harvested at E18.5. Coronal sections from these birth dated *WT* and *Dmrt5*^−/−^ brains were immunostained for Ctip2 and BrdU. The percentage of Ctip2^+^, BrdU^+^, and co-labeled cells was analyzed (*n* = 3, 3, and 3 brains for the three stages; at least three sections of each brain). Examples for co-labeled cells are marked with white arrowheads. Labeled-BrdU nuclei were counted in rectangular fields of 450 μm in width extending from the VZ to the pial surface through the neocortex of *WT* or *Dmrt5*^−/−^ brains. Prevalence of early-born Ctip2 immunoreactive cells was quantified in *WT* and *Dmrt5*^−/−^ brains. (*B*) Quantification of colocalization of the BrdU-labeled cells with Ctip2 among the BrdU^+^ population and (*C*) among the Ctip2^+^ population on at least three sections of each stage injected brains (*n* = 3, 3, and 3). ^*^P < 0.05 (unpaired Student’s *t-test*). Data are given as mean ± SD. There was no significant difference in the numbers of Ctip2^+^ cells in the BrdU+ cohorts labeled at E11.5, E12.5, and E15.5 and examined at E18.5, but there was a significant increase of the proportion of Ctip2^+^ neurons in the cohort of cells labeled by a BrdU pulse at E11.5 but not at E12.5 or E15.5. Scale bars represent 50 μm.

To understand this temporal shift of SPn generation, we analyzed the progeny of the BrdU labeled progenitors with Ctip2/Bcl11, a marker of deep cortical layer neurons, which are born just after SPn. Ctip2/Bcl11 immunoreactive neurons are found in layer Vb and in layer VI (Arlotta et al. 2008; Lennon et al. 2017). Ctip2^+^ neurons contained similarly sized cohorts of birth-dated cortical neurons in *Dmrt5*^−/−^ and in *WT* brains for BrdU injections at E11.5, E12.5, and E15.5 (Fig. 7*A*). However, we observed that more Ctip2^+^ neurons were born at in *Dmrt5*^−/−^ (10%) compared with *WT* (2.8%; ^*^P = 0.03). The proportions in *Dmrt5*^−/−^ and *WT* were similar for BrdU injections at E12.5 and E15.5 (Fig. 7*B*). These data show that in the absence of *Dmrt5*, the Ctip2^+^ dLn was generated earlier than the Nurr1^+^ SPn and that early-born Ctip2^+^ neurons that are normally destined for layer Vb (Arlotta et al. 2005) were positioned abnormally and/or differentiated aberrantly. These results suggest that *Dmrt5* coordinates the timing of emergence of the sequentially generated populations of early-born subcerebrally projecting cortical neurons.

### Radial Migration Defects and Aberrant Multipolar Neuronal Morphology after Loss of *Dmrt5*

**Figure 8:**
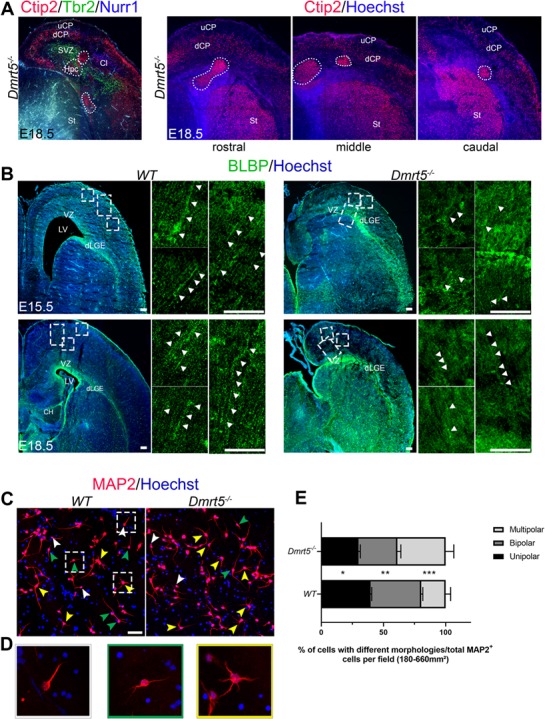
Disturbed radial migration in *Dmrt5*^−/−^ brains and altered morphology of Map2 positive neurons of primary neuronal cultures from *Dmrt5*^−/−^ embryonic brains. (*A*) At E18.5, *Dmrt5*^−/−^ brains show one or more Ctip2^+^ cell containing heterotopias (white dashed circles) in the cortex and Hpc. (*B*) Coronal sections of *WT* and *Dmrt5*^−/−^ E15.5 and E18.5 brains were immunostained with BLBP. BLBP is expressed in CH, radial soma in the VZ, radial glial processes from the VZ to the CP, and in the dorsal most part of the lateral ganglionic eminence (dLGE). High magnification of the neocortex of the boxed areas is depicted in adjacent panels. Radial glial soma in the VZ are present in *WT* and *Dmrt5*^−/−^ brains and the glial fibers observed in *Dmrt5*^−/−^ are oriented similarly as in WT towards the cortical wall (white arrowheads). (*C*) Primary cortical neurons from E18.5 *WT* and *Dmrt5*^−/−^ brains were cultured for 48 h and subsequently immunostained for MAP2 (red) to reveal their projections and counterstained for Hoechst (blue) for their nuclei. Examples of different morphologies identified *in vitro* are depicted with white, green, and yellow arrowheads for unipolar, bipolar, and multipolar neurons, respectively. (*D*) High magnification of the different morphologies is depicted in boxes. Single examples are presented for unipolar, bipolar, and multipolar neurons. (*E*) Quantification of unipolar, bipolar, and multipolar neurons in images from the cultures revealed a significant decrease of unipolar and a significant increase of the multipolar cells *in vitro* (^*^^*^^*^P-value < 0.001; ^*^^*^P-value < 0.01; ^*^P-value < 0.05 (unpaired Student’s *t-test*). Three primary neuronal cultures have been performed on three littermates (two brains for each genotype in each littermate) (a total of MAP2^+^ cells analyzed 978 (*WT*) and 832 (*Dmrt5*^−/−^). dCP: deep CP; dLGE: dorsal most part of lateral ganglionic eminence; St: striatum; SVZ: subventricular zone; uCP: upper CP. Scale bars represent 100 μm in *B* and applies to *A*; scale bar in *C* represents 50 μm.

Similarly to our previous studies, we detected no fundamental change in the expression of non-SP laminar markers between *WT* and *Dmrt5*^−/−^ neocortex (Saulnier et al. 2013). However, we discovered the presence of Ctip2-positive neuronal heterotopias ventrally to layer V in the neocortex of *Dmrt5*^−/−^ embryos (Fig. 8*A*). The disorganized MAP2 and Calretinin expression (Fig. 3*B*,*C*), and the delayed generation and altered distribution of early-born Nurr1 and Ctip2 neurons suggest altered migration that could lead to a smaller and disorganized CP with heterotopias.

We were interested in exploring the mechanisms that led to aberrant cortical migration in *Dmrt5^−/−^* embryos. Radial migration can occur through somal translocation (glia-independent) (Nadarajah et al. 2001), glial-guided migration (Alfano et al. 2011), or multipolar migration (Cooper 2013). The somal translocation of early- and late-born neurons is regulated by the extracellular protein Reelin secreted by Cajal-Retzius cells (Franco et al. 2011; Inoue et al. 2008). Interaction between Cajal-Retzius cells and neurons allows the anchoring of leading process to the MZ and then the movement of neuronal cell bodies along their leading processes (Nadarajah 2003; Nadarajah et al. 2001; Tabata and Nakajima 2003). Similarly to Saulnier et al. (2013), we observed a decrease in *Reelin*^+^ cells in the PP and later in the MZ of *Dmrt5*^−/−^ brains (Fig. 3*A*). MAP2 immunostaining revealed the polarization defects of neuronal cells (Fig. 3*C*), suggesting that the communication between Cajal-Retzius cells and migrating neurons and the attachment of leading processes to the pial surface were impaired.

We examined glial cells and fibers with immunostaining for brain lipid binding protein (BLBP) (Anthony et al. 2004; Kriegstein and Götz 2003; Schmid et al. 2006). At E15.5 and E18.5, BLBP was expressed in the radial glial soma in the VZ of the cerebral wall and in radial glial processes spanning the width of the *WT* cortex (Fig. 8*B*, white arrowheads in high-magnification boxes) (Yu and Zecevic 2011). We also observed a strong BLBP expression in the VZ in *Dmrt5*^−/−^ neocortex. Although fewer radial glial fibers were detected, they were oriented correctly from the VZ to the pial surface of the *Dmrt5*^−/−^ cortex (Fig 8*B*, white arrowheads). BLBP expression was conserved in the lateral migratory stream, over the dorsal most part of the lateral ganglionic eminence (Fig. 8*B*; dLGE) in both *WT* and *Dmrt5*^−/−^ brains. These results suggest that although the RG cells are present in the VZ of Dmrt5^−/−^ cortex, the expansion of radial glial processes is affected. This could contribute to lower efficiency of radial migration and may have altered the cortical development.

We used primary neuronal cultures to analyze the morphology of *Dmrt5*^−/−^ cortical neuronal cells compared with *WT*. We dissected the neocortex of *WT* and *Dmrt5*^−/−^ brains at E18.5, dissociated them, and cultured them for 48 h on poly-D-lysine/laminin coverslips in Neurobasal medium containing B-27 supplement as described in Young et al. (2017) and Muralidharan et al. (2017). The neuronal processes and somatodendritic morphologies were revealed by immunostaining for MAP2 to determine unipolar, bipolar, or multipolar (at least one, two or three processes, respectively) morphologies (Fig. 8*C*,*D*). We observed a lower proportion of bipolar and a higher proportion of multipolar cells in *Dmrt5*^−/−^ (38.9 ± 6.52%) cortical cultures compared with the *WT* (19.6 ± 3.98%) (^*^^*^^*^P-value <  0.001) (Fig. 8*E*). Thus, the transition between multipolar to bipolar morphology of migrating neurons was affected in *Dmrt5*^−/−^ cultures.

## Discussion

Transcription factors are intrinsic regulators for the decision of NPs to proliferate or differentiate (Britanova et al. 2005; Nieto et al. 2004; Tarabykin et al. 2001). Increasing or decreasing the level of these proteins disrupts the balance between progenitor self-renewal and differentiation and can lead to changes in the thickness of the mature cortex (Caviness et al. 2003; Chenn and Walsh 2003, 2002; Englund et al. 2005; Rakic 1995). In the present study, we showed that *Dmrt5* plays an important role in the dynamics of basal progenitors and transitioning IPCs, affecting the timing of early-born neuron (SP and dLn) generation. Our study also revealed that Dmrt5 is expressed in postmitotic SPns as well as some cortical migrating neurons and may be involved in the switch of multipolar to bipolar cortical neuronal migration mode. These observations provide a better understanding of the underlying mechanisms that are involved in cortical thickness reduction in *Dmrt5*^−/−^ embryos and of the microcephaly in human (De Clercq et al. 2018; Desmaris et al. 2018; Konno et al. 2012; Saulnier et al. 2013; Urquhart et al. 2016).

In our study, we show that the loss of *Dmrt5* in the lateral cortex reduces the number of Tbr2^+^ IPCs and increases the proportion of double positive Pax6^+^Tbr2^+^ cells. Assuming *Drmt5* disruption does not affect the timing of the transition from Pax6^+^Tbr2^−^ through Pax6^+^Tbr2^+^ to Pax6^−^Tbr2^+^ progenitors, these results indicate an increase of transitioning from RGCs to IPCS. However, as the proportion of Tbr2^+^ IPCs decreases, we could hypothesize that Dmrt5 regulates the time-course of Pax6 expression loss and/or Tbr2 expression gain of transitioning progenitor cells and they stay in the transitioning state for longer. Tbr2 is known to regulate the division and neurogenesis of IPCs (Arnold et al. 2008; Bayatti et al. 2008; Bulfone et al. 1999; Englund et al. 2005; Mihalas et al. 2016; Vasistha et al. 2015). The reduction of the thickness of the lateral cortical wall of *Dmrt5*^−/−^ brains could thus be due to the depletion of IPCs and downstream of a decrease in neuronal production. Effects on the spindle orientation of progenitors are also well known to influence the choice between direct or indirect neurogenesis (Postiglione et al. 2011).

In this study, we show that Dmrt5 is detectable by immunofluorescence not only in progenitors but also in some postmitotic cells including SPn. This may be due to the prolonged stability of the protein as ISH does not show *Dmrt5* in SP while it does in CR cells (Konno et al. 2012; Saulnier et al. 2013). *Dmrt5* mRNA was not detected to be enriched in laser microdissected SP and lower cortical neurons of E15.5 embryos (Oeschger et al. 2012). Dmrt5 immunoreactivity in these postmitotic cells was observed enriched outside the nucleus. This suggests that the nuclear import of Dmrt5 may be under differential regulation in postmitotic and progenitor cells. Regulation of nuclear trafficking has been shown to be important in the control of the activity of other transcription factors (Zhang et al. 2002). Whether this is also the case for Dmrt5 remains to be explored.

Our results confirmed the incomplete formation of the SP and accelerated neurogenesis in *Dmrt5*^−/−^ embryos that was previously observed (Saulnier et al. 2013; Young et al. 2017). This loss of SP occurs despite an increase of dLn generation, suggesting that Dmrt5 is required to specify SP fate. Cortex and the RMTW are the two major sources of glutamatergic SPns. Since Dmrt5 is expressed in the germinal zones of both regions, it is conceivable that SPns with cortical and RMTW origins are both affected in *Dmrt5*^−/−^ brains. RMTW is probably a minor contributor to SP because only a relatively small population of SP cells are generated at E10.5 (Hoerder-Suabedissen and Molnár 2013) and RMTW-derived SP cells are known to be generated at E10 and E11 (Pedraza et al. 2014). Our study shows that the loss of *Dmrt5* has the greatest impact at E11/E12 and later stages on SP generation, suggesting that the majority of DMRT5^+^ SP cells are from the cortical VZ/SVZ source. Moreover, while *Wnt2b* and *Wnt3a* expressions in the cortical hem (CH) are dramatically reduced in *Dmrt5*^−/−^, *Fgf17* in the pallial septum containing, the RMTW appears rather unaffected (Desmaris et al. 2018). This supports the cortical VZ/SVZ as the source of the remaining SPn observed in *Dmrt5*^−/−^ and *Dmrt3*^−/−^ caudal cortex.

Our results also indicate that *Dmrt5* expression in cortical progenitors is required for SP fate specification during a short action window (E9.5–E10.5). Indeed, in *Dmrt5^lox/lox^*;*Emx1^Cre^*, the residual expression of *Dmrt5* before its ablation allows the generation of SPn as observed at E18.5. Moreover, the *Dmrt5* ablation after E10.5 in *Dmrt*5^lox/lox^;Nestin*^Cre^* has no impact on SP either at midgestation or at later stages. Whether Dmrt5 plays a role in postmitotic CR cells and SPn is not known. This question could be addressed using *Nex^Cre^* induced *Dmrt5* inactivation and remains to be addressed.

Unexpectedly, in *Dmrt5*^−/−^ brains, some SPns are still present in the caudomedial cortex where *Dmrt5* has the highest expression. We previously reported the redundant function of *Dmrt3* and *Dmrt5* in the cortex (Desmaris et al. 2018). This redundancy between *Dmrt3* and *Dmrt5* could explain the presence of residual SPn observed at later stages in the caudal part of the brain of *Dmrt5*^−/−^ and *Dmrt3*^−/−^ embryos. *Dmrt4* is expressed in a gradient opposite to that of *Dmrt3* and *Dmrt5* and upregulated in *Dmrt3* and *Dmrt5* mutants, (De Clercq et al. 2018). Whether *Dmrt4* responsible of the persistence of some SPn and CR cells remains also to be investigated.

Neuronal migration is also altered in the cortex of *Dmrt5*^−/−^ embryos. This is likely to be due, at least in part, to incomplete PP splitting and reduction in Cajal-Retzius cells in the MZ, as observed in *Sox5* mutants (Kwan et al. 2008; Lai et al. 2008).

Our study revealed the defects in polarization of neuronal processes in *Dmrt5*^−/−^ neurons, suggesting that the interaction between Cajal-Retzius and SP cells and early-born cortical neurons may also be altered. The disruption in cell adhesion molecule (*N*-Cadherin) to attach the glial fibers or defects in the endocytosis/recycling processes and nuclear elongation, which are known to cause altered radial migration (Rakic 1988; Shikanai et al. 2011), should thus be investigated. A recent study by Ohtaka-Maruyama and collaborators has shown that the SP is required for the multipolar to bipolar morphology switch of migrating neurons (Ohtaka-Maruyama et al. 2018). This raises the possibility that the altered mode of neuronal migration in the cortex of *Dmrt5*^−/−^ embryos is a secondary consequence of the reduction of the SP.

Together, our analysis suggests that the loss of *Dmrt5* in apical progenitors leads to defects in neurogenesis, altered split of PP, and defects in SP, and disturbed radial cortical migration. This abnormality has specific time and regional sensitivity. The altered SP formation could further contribute to the increased number of multipolar neurons, and subsequently the slowdown of neuronal migration and disorganization of the cortical wall.

## Funding

Collaborative grant from the Wiener—Anspach Foundation to E.J.B. and Z.M. (Role of the Dmrt5 Transcription Factor in the Development of the Earliest Cortical Circuits); work in the laboratory of E.J.B was supported by grants from the Fund for Scientific Research (FRFC 6973823, CDR 29148846); Walloon Region (First International project “NEURON”); Jean Brachet Foundation; work in the laboratory of Z.M. was funded by Medical Research Council (UK), (G00900901, MR/N026039/1); Royal Society and Anatomical Society. Work in the laboratory of T.T. was supported by the Medical Research Council (MR/K013750/1).

## Notes

We thank members of the Center for Microscopy and Molecular Imaging (CMMI), which is supported by the Hainaut-Biomed FEDER program, C. Chevalier for technical assistance. L.R. is a BEWARE postdoctoral fellow from the Walloon Region; E.D is a Wallonie-Bruxelles International (WBI) doctoral fellow from the Wallonia-Brussels Federation. F.G.-M. held an HFSP Fellowship at the Department of Physiology, Anatomy and Genetics, University of Oxford, Oxford, UK.

*Conflict of Interest:* None declared.

## Supplementary Material

FigS1_Ratie_et_al_bhz310

FigS2_Ratie_et_al_bhz310

FigS3_Ratie_et_al_bhz310

FigS4_Ratie_et_al_bhz310

FigS5_Ratie_et_al_bhz310

Ratie_et_al_Supplementary_Figure_Legends_bhz310
