# Supplementary figures and images for: Loss of Dmrt5 Affects the Formation of the Subplate and Early Corticogenesis
Source: Cereb Cortex. 2019 Dec 16;30(5):3296–312. doi: 10.1093/cercor/bhz310 (PMC7197206; doi:10.1093/cercor/bhz310)

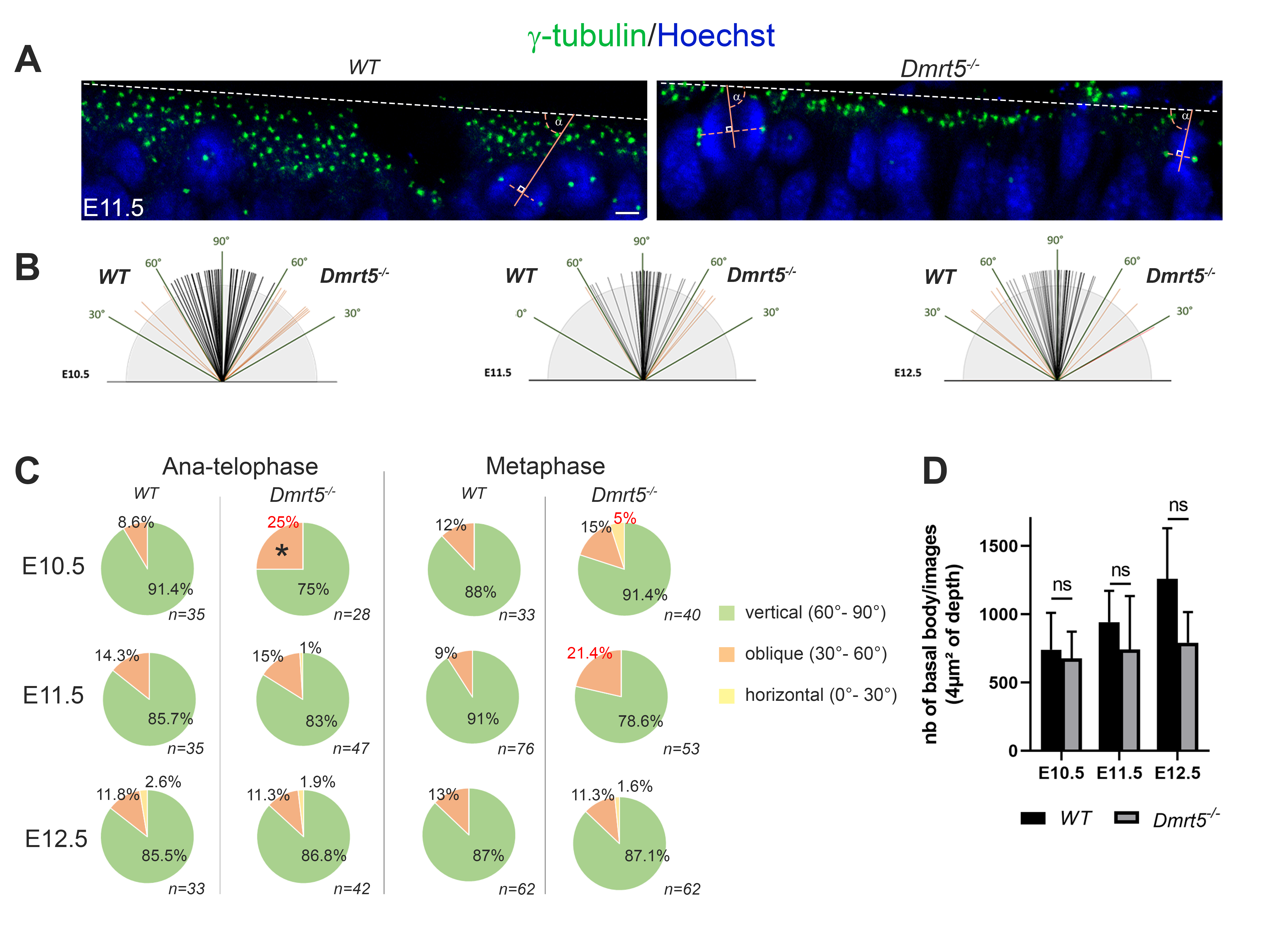

Supplement: FigS1_Ratie_et_al_bhz310 [file figs1_ratie_et_al_bhz310.png]

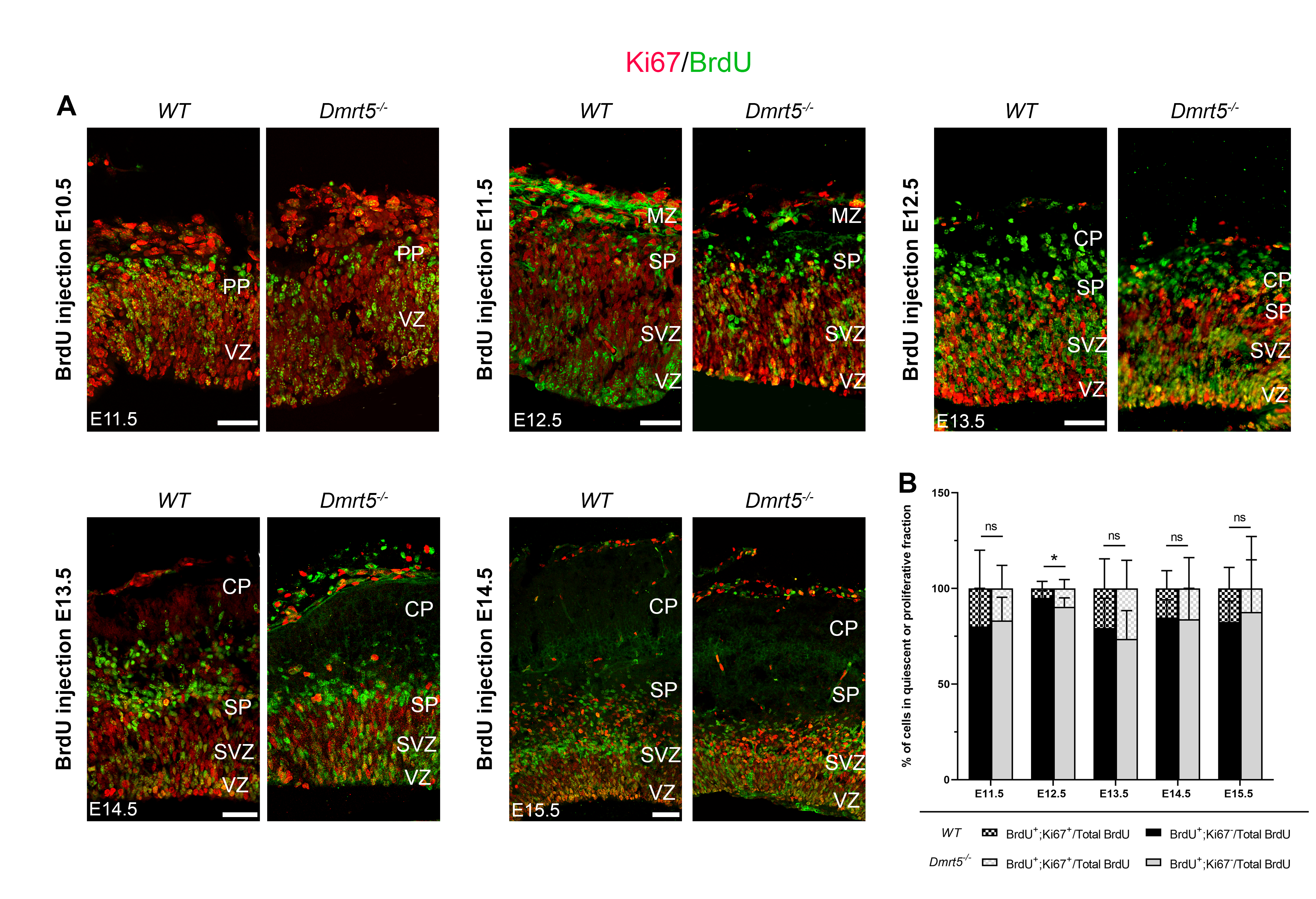

Supplement: FigS2_Ratie_et_al_bhz310 [file figs2_ratie_et_al_bhz310.png]

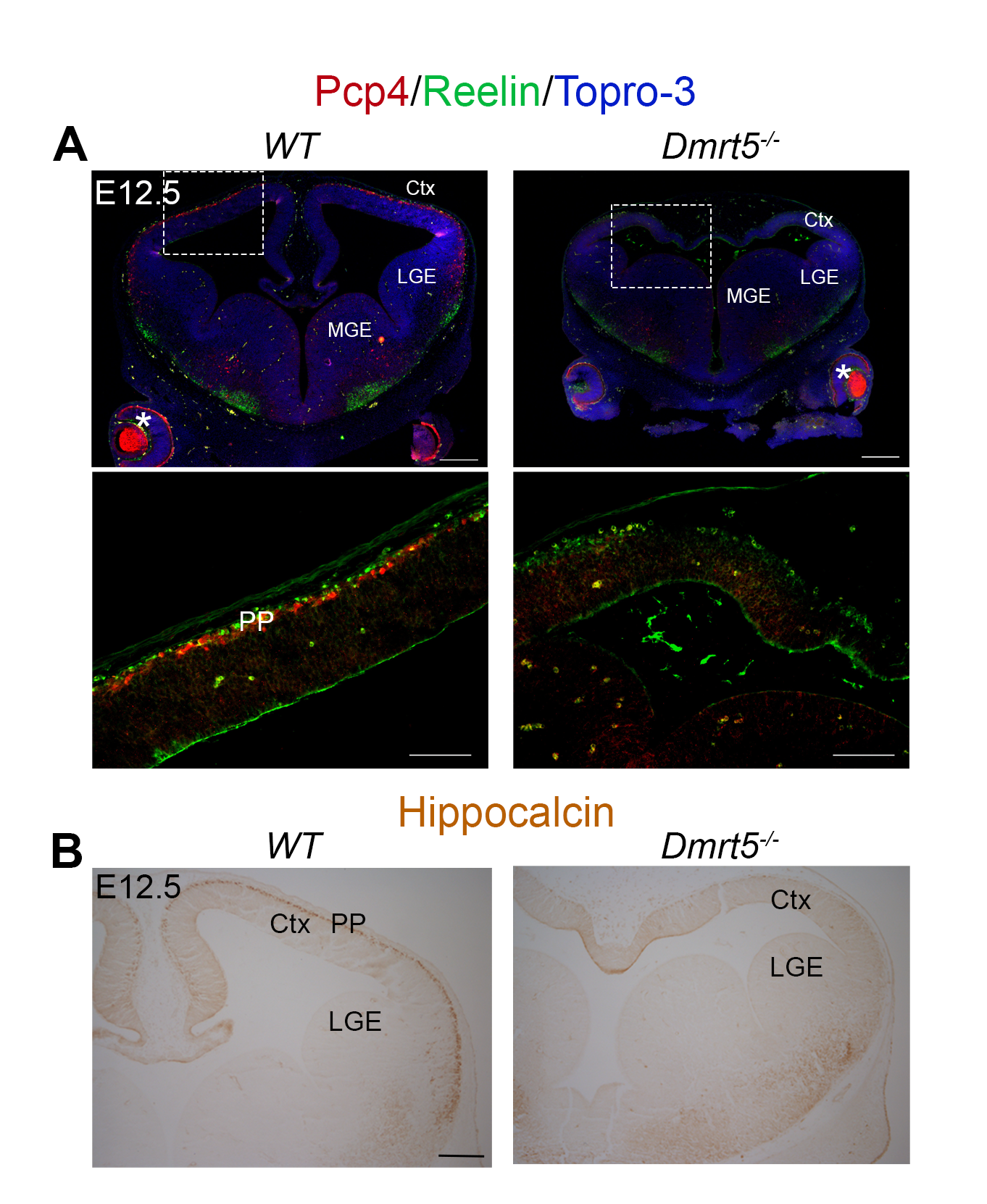

Supplement: FigS3_Ratie_et_al_bhz310 [file figs3_ratie_et_al_bhz310.png]

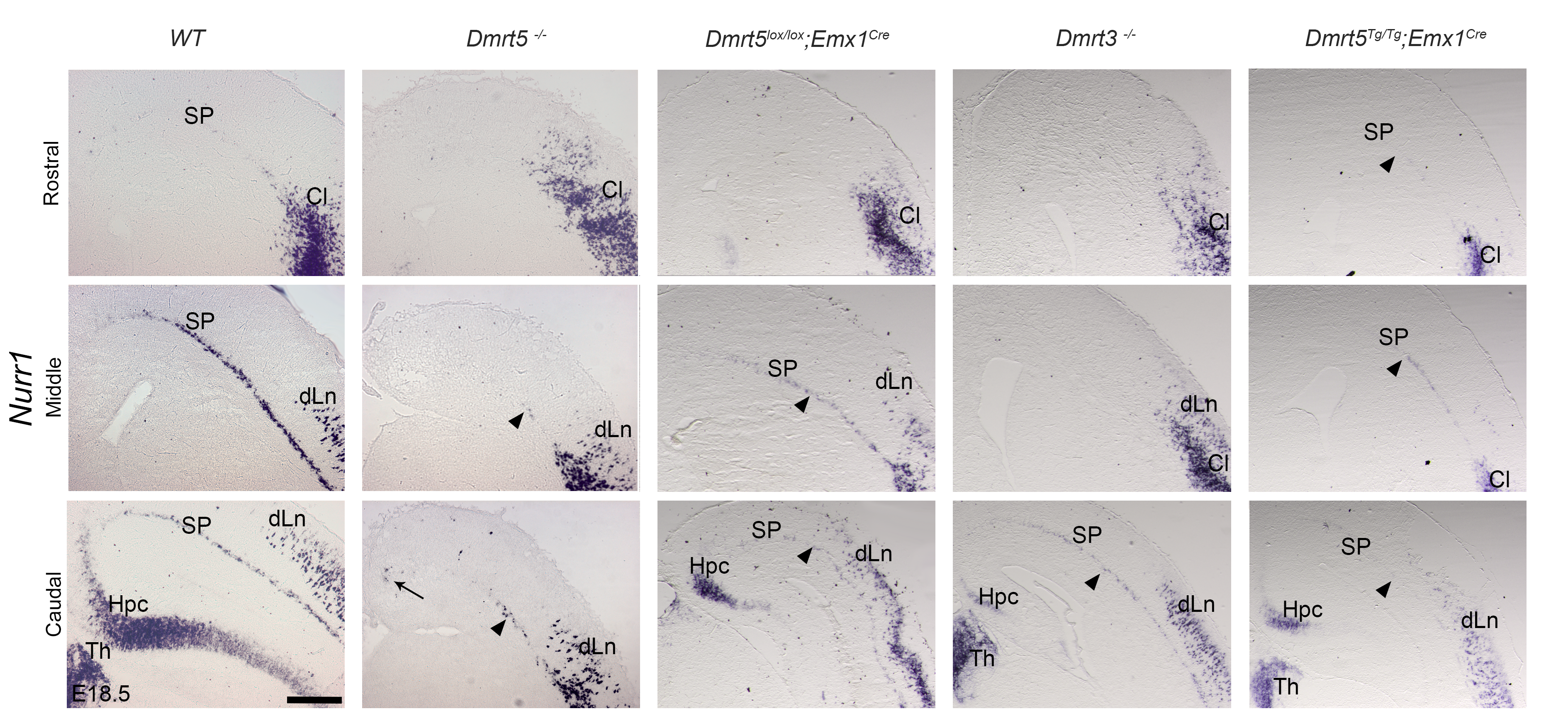

Supplement: FigS4_Ratie_et_al_bhz310 [file figs4_ratie_et_al_bhz310.png]

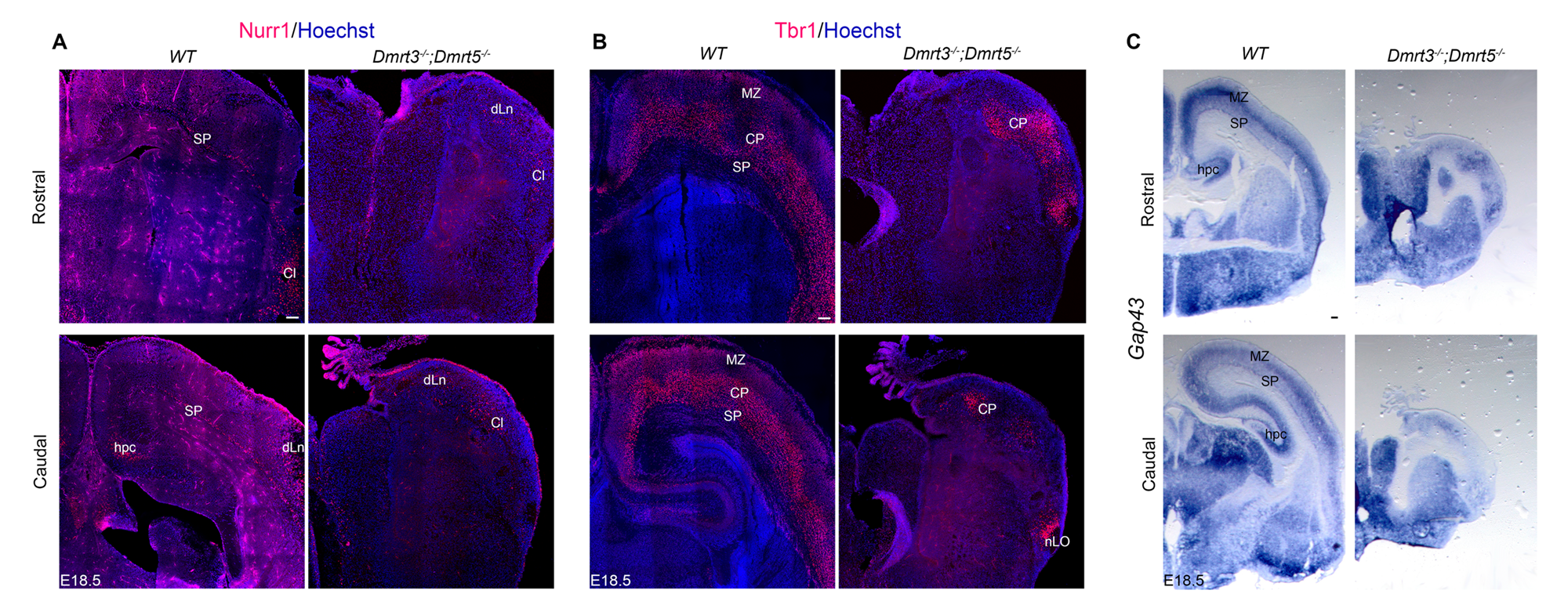

Supplement: FigS5_Ratie_et_al_bhz310 [file figs5_ratie_et_al_bhz310.png]
