# Supplementary material for: Loss of Dmrt5 Affects the Formation of the Subplate and Early Corticogenesis
Source: Cereb Cortex. 2019 Dec 16;30(5):3296–312. doi: 10.1093/cercor/bhz310 (PMC7197206; doi:10.1093/cercor/bhz310)
Supplement: Ratie_et_al_Supplementary_Figure_Legends_bhz310 [file ratie_et_al_supplementary_figure_legends_bhz310.docx]

**Figure S1. Mitotic spindle orientation of apical progenitor cell division in *Dmrt5*^-/-^ cortex is altered at E10.5, but no significantly different at E11.5 and E12.5.**(A) Cleavage angles of progenitor cells at the apical surface in *WT* and *Dmrt5*^-/-^ embryos were measured at E10.5, E11.5 and E12.5. Coronal sections are immunostained with γ-tubulin antibody to detect centrosomes (green) and angles (see angle indicated with dotted lines) are measured in metaphase and in anaphase-telophase when the final division axis is established. (B) Distribution of division plane in *WT* and *Dmrt5*^-/-^ sections at E10.5, E11.5 and E12.5 (60°-90°: vertical, 30°-60°: oblique and 0°-30°: horizontal). The angle distribution was not significantly different between *WT* and *Dmrt5*^-/-^ at E10.5, E11.5 and E12.5. (C) Diagram representing the percentage of cleavage angle groups at ana-telophase and metaphase for embryos at E10.5, E11.5 and E12.5. The spindles of most apically dividing cells at ana-telophase are oriented along a horizontal plane (the spindle axis is horizontal to the ventricular apical surface and the cleavage furrow positions orthogonally to the ventricular lining). The proportion of oblique division significantly increases at E10.5 in *Dmrt5*^-/-^ brains (25%) compared to *WT* brains (8.6%) in ana-telophase (*, p< 0.05). We observed an increase of oblique division at metaphase, implying that the spindle orientation could be affected prior to chromosome segregation but rescued at mitotic exit (ns, p= 0.37). At E11.5 and E12.5, the proportions of different cleavage angles are conserved between *WT* and *Dmrt5*^-/-^ brains as well as E13.5 (data not shown). (D) We used γ-tubulin staining to reveal the basal body to compare the number of cilium base in *WT* and *Dmrt5*^-/-^ cortex at E10.5, E11.5 and E12.5, but we did not find significant differences. Scale bar 5 µm (A).

**Figure S2. The proliferating behaviour of cortical progenitors is transiently affected at E12.5 in *Dmrt5*^-/-^ embryos.**

(A) To determine cortical progenitor cell cycle exit rate in *WT* and *Dmrt5*^-/-^ embryos, we combined Ki67 immunohistochemistry and BrdU birthdating. The mitotic marker Ki67 is a nuclear protein that is expressed in all phases of the cell cycle except the resting phase. We gave pulse labelling of BrdU at different stages (E10.5, 11.5, 12.5, 13.5 and 14.5) and brains were collected 24h after the pulse labelling, fixed, and coronal sections were stained for Ki67 and BrdU immunoreactivity. BrdU^+^Ki67^-^ cells are cells that were dividing at the time of BrdU injection but have exited the cell cycle by the time of analysis. (B) A 5% decrease in the number of cells leaving the cell cycle (quiescent fraction, filled graph, 95.1% vs 90.4%, * p<0.05, unpaired Student’s) and a corresponding increase in the fraction of cells remaining in the cell cycle (proliferative fraction, striped graph, 4.6% vs 9.6%, *p<0.05, unpaired Student’s) was observed at E12.5 following BrdU injection at E11.5 in the cortex of *Dmrt5*^-/-^embryos. Note that BrdU-positive cell distribution in *Dmrt5*^-/-^ is less homogenous at early stages along the cortical depth than in *WT*. A greater fraction of BrdU^+^ cells is observed in upper layers while fewer are found in deeper layers compared to *WT*. At subsequent stages, the proportions of cells in the quiescent and proliferative fractions are similar.

CP : cortical plate; dpL: deep layers; MZ: marginal zone; PP: preplate; SP: subplate; SVZ; subventricular zone; upL: upper layers; VZ: ventricular zone

Scale bars represent 50 µm for all images.

**Figure S3. Pcp4 and Hippocalcin expression is strongly reduced in the *Dmrt5*^-/-^ brains.**

(A) Coronal sections of E12.5 heads from *WT* and *Dmrt5*^-/-^ embryos were immunostained for Pcp4 and Reelin (A) and Hippocalcin (B) at E12.5. Pcp4^+^ (red) and Reelin^+^ (green) cells were detected in the preplate of *WT* embryos. Enlarged images of dorsal cortex from the regions indicated with boxes demonstrate the lack of Pcp4^+^ cells in PP where a few Reelin^+^ cells continue to be detected in *Dmrt5*^-/-^. In contrast to the cortex, Pcp4 expression is conserved in the retina of *Dmrt5*^-/-^ embryos (asterisks). (B) We observed strong reduction of hippocalcin immunoreactivity in the PP of *Dmrt5*^-/-^ compared to WT at E12.5. Scale bars represent 250 µm and 100 µm for high magnification boxes of Pcp4 and Reelin. Ctx: Cortex; LGE: lateral ganglionic eminence; MGE: medial ganglionic eminence; PP: preplate.

**Figure S4.** **Subplate defects in the cortex of *Dmrt3*^-/-^ , *Dmrt5^lox^*^/lox^;*Emx1^Cre^*** **and of *Dmrt5^Tg/Tg^;Emx1^Cre^* embryos.**

*In situ* hybridization for Nurr1 in rostral, middle and caudal coronal sections from *WT*, *Dmrt5*^-/-^, *Dmrt5^lox^*^/lox^;*Emx1^Cre^*, *Dmrt3*^-/-^ and *Dmrt5^Tg/Tg^*;*Emx1^Cre^* embryos at E18.5.
Scattered *Nurr1*^+^ SP cells were observed in the middle and caudal regions of the *Dmrt5*^-/-^ brains (black arrowheads). Few *Nurr1*^+^ cells were detected in the hippocampal anlage (black arrow in caudal section of *Dmrt5*^-/-^). *Nurr1* expression in subplate was less disturbed in *Dmrt5^lox^*^/lox^;*Emx1^Cre^* and *Dmrt3*^-/-^ brains than in *Dmrt5*^-/-^ (black arrowheads) and appears normal in *Dmrt5* overexpressing embryos.

Scale bar represent 250 µm for all panels.

Cl: claustrum; dLn: deep layer neurons; Hpc: hippocampus; SP: subplate; Th: thalamus.

**Figure S5: Subplate layer is not detected, but claustrum is still present in *Dmrt3*^-/-^; *Dmrt5*^-/-^ double mutant brains at E18.5.**

(A,B) Coronal sections of developing brains from *WT* and *Dmrt3*^-/-^;*Dmrt5*^-/-^ E18.5 embryos immunostained for Nurr1 (A) and Tbr1 (B). Scattered Nurr1^+^ cells are present in the remaining deep layers and in the claustrum in *Dmrt3*^-/-^;*Dmrt5*^-/-^ brains but the subplate layer is not detected even in caudal parts of the brain. Tbr1 is detected in the residual cortical plate of *Dmrt3*^-/-^;*Dmrt5*^-/-^ embryos but no separate subplate layer is observed. Tbr1 expression in the MZ is also not detected in *Dmrt3*^-/-^;*Dmrt5*^-/-^ embryos. (C) Coronal sections of developing brains from *WT* and *Dmrt3*^-/-^;*Dmrt5*^-/-^ E18.5 embryos *in situ* hybridisation labelled for *Gap43*. *Gap43* is detected in marginal layer, the cortical plate and the subplate in *WT* brains whereas the *Gap43* staining revealed the disorganized cortex in *Dmrt3*^-/-^; *Dmrt5*^-/-^ brains.

Scale bars represent 100 µm for all images.
